# Bacterial Infections Role in Gynecological Cancers Development: Narrative Review

**DOI:** 10.1002/cnr2.70499

**Published:** 2026-04-12

**Authors:** Robab Azargun, Maryam Azargoon, Zahra Asefy, Mina Yekani, Vahideh Tarhriz, Fatemeh Yeganeh, Mohammad Yousef Memar, Shirin Eyvazi

**Affiliations:** ^1^ Medicinal Plants Research Center Maragheh University of Medical Sciences Maragheh Iran; ^2^ Department of Knowledge and Information Science Shahid Chamran University of Ahvaz Ahvaz Iran; ^3^ Pediatric Health Research Center Tabriz University of Medical Sciences Tabriz Iran; ^4^ Cardiovascular Center of Excellence, Louisiana State University Health Sciences Center New Orleans Louisiana USA; ^5^ Microbiology Department, School of Medicine Tabriz University of Medical Sciences Tabriz Iran; ^6^ Infectious and Tropical Diseases Research Center Tabriz University of Medical Sciences Tabriz Iran; ^7^ Department of Immunology Mayo Clinic Phoenix Arizona USA

**Keywords:** bacterial infection, *Chlamydia*, gynecological cancers, microbiota, *Mycoplasma*, reproductive organs

## Abstract

**Background:**

Gynecological cancers are among the most common cancers in women that affect female reproductive organs. The most common gynecological cancers are ovarian, cervical, uterine/endometrial, vaginal, and vulvar cancer. Women's reproductive organs have a dynamic and relative microbial balance. The disruption in the balance of the microbiome could result in numerous gynecological diseases, as well as, gynecological cancers. In this study, we aimed to review new findings on the role of different bacterial infections in various types of gynecological cancers.

**Recent Findings:**

The role of bacterial infection, as an external factor, has been established in several cancers. However, the ways in which bacteria can promote the development of cancer are not fully understood. It seems that inflammation induced by bacterial infections could promote carcinogenesis. In addition, bacterial toxins and effector proteins play important roles in the progression of cancer. In this review, we attempt to present the different bacterial infections, which have been linked to gynecological cancers development. According to different researches, *Chlamydia*, *Mycoplasma*, and *Bacteroides* spp. are the most common bacterial infections associated with gynecological cancers.

**Conclusion:**

Evaluation of microbiome in reproductive organs of the patients with gynecological cancer and studies on prevention and control of the infections in the patients could be useful in verification of pathogenesis of the diseases and also founding suitable therapeutic interventions.

## 1 Introduction

Gynecological cancers are some of the most common cancers in women, originating from the reproductive organs [1, 2]. These malignancies are the fourth most frequently diagnosed neoplasms in women of childbearing age, accounting for 16% of all neoplasms [3]. The main types of gynecological cancers are cervical, ovarian, uterine (endometrial cancer and uterine sarcoma), vaginal, and vulvar cancers [4]. The etiology of gynecological cancers is multifactorial; genetic and epigenetic factors, environmental factors, and infection are the most common risk factors for these malignancies [5]. Recently, the role of the microbiome in the development of various diseases has been well established [6, 7]. Microbes are involved not only in the onset of gynecologic cancer but also emerge as a result of the physiological disruptions it causes [8, 9]. During dysbiosis, characterized by an imbalance of commensal and pathogenic microorganisms [6], the reduction and absence of *Lactobacillus* is often observed and replaced by specialized or facultative anaerobic bacteria, compromising the vaginal immune defense [10]. Decreased estrogen levels are associated with vaginal dysbiosis, characterized by a reduction of regular hydrogen peroxide‐ and lactic acid‐producing *Lactobacillus* species in the vagina and an overgrowth of anaerobic bacteria such as Gardnerella, Mycoplasma, and Prevotella. This shift leads to an increase in vaginal pH above 4.5. Anaerobic bacteria like *Gardnerella vaginalis* possess virulence factors that allow them to adhere to the host epithelium and form a biofilm [11]. Dysbiotic bacterial communities release enzymes such as sialidase that compromise the integrity of the mucus barrier and damage the cervicovaginal epithelium, increasing the susceptibility of basal cells to HPV infection. Additionally, certain bacterial toxins can cause DNA damage in host cells, promoting the integration of viral oncogenes into the host genome [12].

The association between viral infections—particularly Human Papillomavirus (HPV) and Human Immunodeficiency Virus (HIV)—and the development of cervical cancer is well established in the literature [13, 14, 15].

Additionally, chronic infections resulting from PID can contribute to the release of tumor‐promoting factors such as cytokines, chemokines, and reactive oxygen species, fostering genetic and epigenetic changes associated with carcinogenesis [16].

For example, bacterial infections localized in the peritoneum and vaginal infections (such as *Neisseria gonorrhoeae* or *Chlamydia trachomatis* ) may contribute to the progression and metastasis of ovarian cancer. This may be due to inflammation‐induced oxidative stress, which leads to the accumulation of DNA damage and mutations [17].

However, the role of bacterial infections in these malignancies has received less attention. Bacteria colonize various human organs and, beyond causing infection‐related conditions, they are implicated in numerous disorders such as obesity, diabetes, fatty liver disease, allergic diseases, atherosclerosis, autoimmune diseases, Alzheimer's disease, and cancer [18, 19, 20, 21, 22, 23, 24, 25, 26]. The association between bacterial infections and cancer development is complex and not yet fully understood. However, genetic susceptibility and inflammation appear to be key mediators in this association. In this study, we aim to present new findings on the role of different bacterial infections in various types of gynecological cancers by reviewing recent literature.

## 2 Methodology

In this review, data on the role of bacterial infections in the development of gynecological cancers were collected from databases including PubMed, Scopus, and Google Scholar. Published manuscripts were searched using keywords such as bacterial infections, gynecological cancers, and microbiome in reproductive organs. All English‐language articles were screened and independently read by two authors. An overview of the literature search strategy, including inclusion/exclusion criteria and results, is provided in Figure S1.

## 3 The Involvement of Bacteria in Cancer Development

Promotion and progression of cancer is a complex multistage process, which is accompanied by genetic alterations. Genetic alterations can be induced by various internal and external agents. The role of bacterial infection as an external factor has been established in several cancers [24, 25, 26, 27]. For instance, it has been known that *Helicobacter pylori* ( *H. pylori* ) infection could be associated with gastric cancer promotion [26, 28]. World Health Organization (WHO) defined *H. pylori* as class I carcinogens [28]. The other well‐known bacterial infections are chronic *Salmonella typhi* ( *S. typhi* ) infections, which are associated with gallbladder cancer, and *Salmonella enteritidis* associated with colon cancers [29]. Other bacteria related to cancer promotion in different organs are listed in Table 1. Investigations in germ‐free and antibiotic‐treated animals showed that bacteria can promote cancer in different experimental systems such as colon and liver cancers [30, 31]. The mechanisms by which bacteria can promote development of cancers are not fully addressed. However, several likely mechanisms have been suggested, which are categorized into bacterial cell‐surface components, bacterial toxins, and effector proteins. These components are derived from chronic inflammation, which may change normal physiological processes and result in genomic instability and promotion of cancer development [24, 25, 32]. For example, it has been shown that the high level of adhesion A (fadA) of *Fusobacterium nucleatum* is linked to overexpression of Wnt signaling pathway genes, which are involved in inflammation and tumorgenicity [24, 33]. In addition, CagL of *H. pylori* increases gastric secretion and is linked to hypergastrinemia, which may lead to gastric cancer [34]. CagL is found in *H. pylori* strains that carry the type IV secretion system (T4SS), which is encoded by the Cag pathogenicity island and recognized as the main virulence factor of this bacterium. CagL is located at the tip of the T4SS, where it acts as a specialized adhesin that binds and activates the α5β1 integrin receptor on gastric epithelial cells through its arginine‐glycine‐aspartate (RGD) motif, thereby promoting the injection of the bacterial oncoprotein CagA into host cells [35, 36]. The interaction between the *H. pylori* T4SS and host integrin α5β1 can trigger the activation of NF‐κB signaling and induce the release of key pro‐inflammatory cytokines. This can lead to more severe clinical outcomes, including gastric carcinogenesis [37]. Recent studies have also shown that CagL can stimulate the expression of ADP‐ribosyltransferase, NAD‐glycohydrolase, and auto‐ADP‐ribosylation activities. This highlights its potential as a candidate for *H. pylori* vaccine development [38].

**TABLE 1:** Different bacterial infections related to cancer promotion in different gynecological organs.

Lung cancer	Gastric cancer	Colorectal cancer	Esophageal cancer	Gallbladder cancer	Breast cancer
*Mycoplasma*	*Helicobacter pylori*	*Bacteroides fragilis strains*	*Fusobacterium nucleatum*	*Salmonella Typhi*	*Clostridia*
		*Escherichia coli*	*Escherichia coli*		*Ruminococcaceae families*
		*Streptococcus bovis*			

Another example is the blood group antigen‐binding adhesin BabA of *H. pylori* , which is related to high levels of inflammatory cytokines and interleukins such as CCL5 and IL‐8. In addition, bacterial protein may increase cancer‐related genes such as CDX2 and MUC2 involved in colorectal cancer [39]. Furthermore, lipopolysaccharides (LPS), a bacterial cell‐surface component, activate host Toll‐like receptor‐4 (TLR‐4), which is related to several inflammatory pathways. The association of TLR4 activation and cancer has been identified in a mice model of colorectal cancer. It has been shown that TLR4 is overexpressed in colorectal patients [24, 40].

Several bacterial products such as toxins and metabolites cause DNA damage and dysregulation of key factors, which are related to the host cell cycle and cell division regulation. For instance, cytolethal distending toxin (CDT), which is produced by numerous Gram‐negative bacteria such as *S. typhi* , *E. coli* , and *Shigella dysenteriae*, induces double stranded DNA (ds DNA) breaks in the host genome. The role of the toxin is studied well in gallbladder cancer [41]. CDT also promotes mitogen‐activated protein kinase (MAPK) activity, which is related to oncogenic pathways [42]. In addition to CDT, colibactin toxin is secreted by group B2 *E. coli* strains harboring the polyketide synthetase (pks) island, induces ds DNA breaks in the host genome and leads to tumorigenesis in the host cells [43]. Furthermore, several bacterial toxins induce tumorigenesis by increasing and decreasing cell proliferative and cell death factors. CagA of *H. pylori* activates c‐Met receptor of epithelial cells, which is a proliferative factor of the cells. In addition, CagA can induce tumorigenesis of gastric epithelial cells via interaction with different cancer‐related pathways including MEK, ERK, and β‐catenin pathways [29, 44, 45]. Enteric *Salmonella* effector AvrA intervenes with different colon cell signaling pathways, changes immune response, apoptosis, and cell proliferation [46]. Overall, pathogenic bacteria manipulate the host cells' niche through different mechanisms, and the identification of these mechanisms can be useful in combating them.

## 4 The Potential of Bacteria in Development of Gynecological Cancers

**FIGURE 1:**
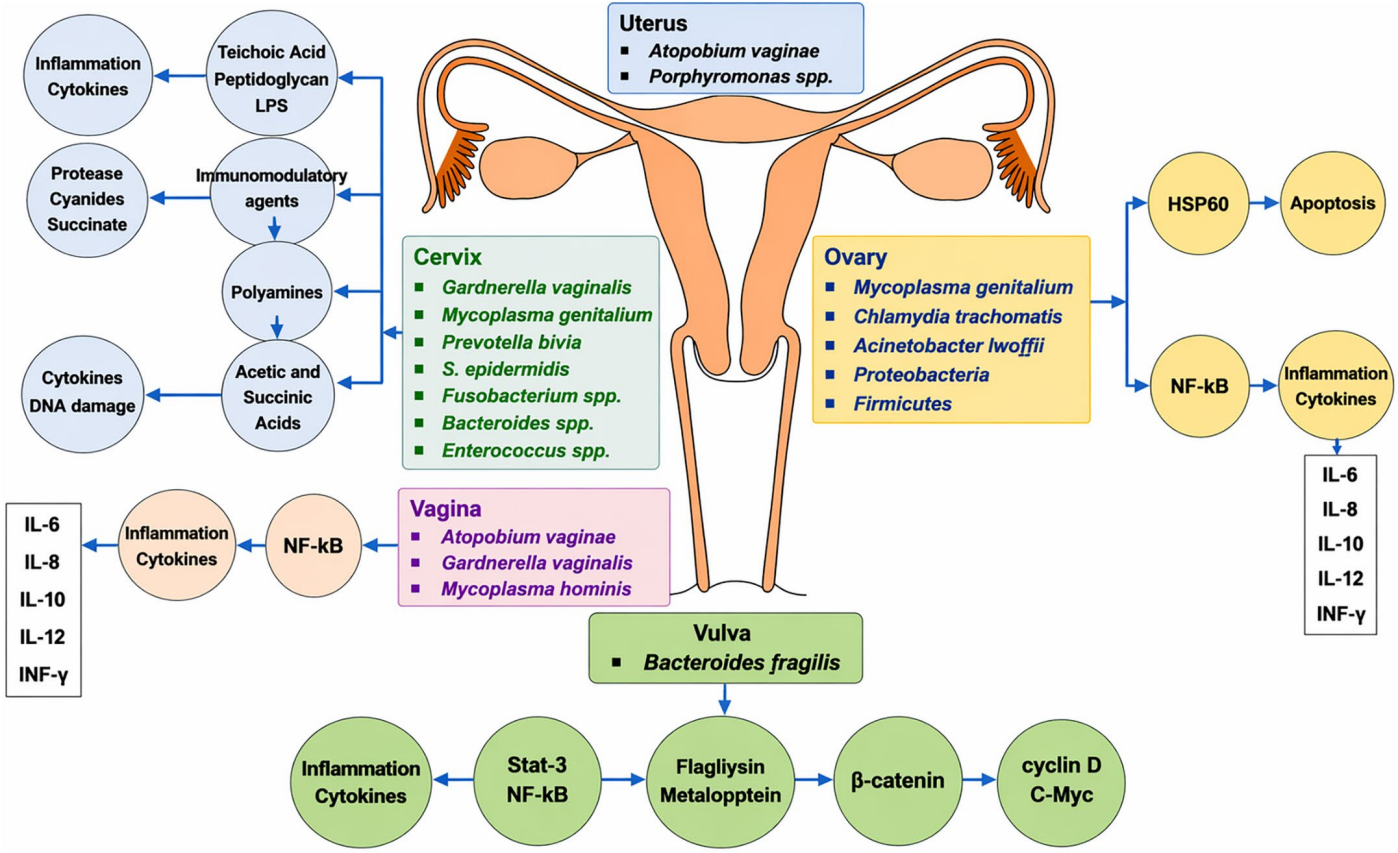
The different bacteria and their induced mechanisms associated with various gynecological organs.

Women's reproductive organs have a dynamic and relative microbial balance. Gynecologic cancers originate in the reproductive organs of women, commonly affecting the cervix, endometrium, and ovaries, while cancers of the vagina and vulva are less frequent. Alterations in the gut and vaginal microbiome composition influence the immune and metabolic signaling of host cells, leading to chronic inflammation, angiogenesis, cellular proliferation, genome instability, breaches in the epithelial barrier, and metabolic dysregulation that may trigger the progression of gynecologic cancers. An imbalance in the microbiome, as well as, gynecological cancers. The association of different types of gynecological cancers including cervical, ovarian, uterine (endometrial cancer and uterine sarcoma), vaginal, and vulvar cancers with different bacterial infections has been investigated in several researches which are discussed in the following sections. The most important bacteria associated with different types of gynecological cancers and the related mechanisms are summarized in Table 2 and Figure 1, respectively.

**TABLE 2:** The different bacterial infections associated with gynecological cancers development.

Ovarian cancer	Uterine/endometrial cancer	Cervical cancer	Vulvar cancer	Vaginal cancer
*Chlamydia trachomatis*	*Atopobium vaginae*	*Gardnerella vaginalis*	*Bacteroides Fragilis*	*Gardnerella vaginalis*
*Mycoplasma genitalium*	*Porphyromonas* sp.	*Mycoplasma genitalium*	*Chlamydia trachomatis*	*Mycoplasma hominis*
*Proteobacteria*		*Prevotella bivia*		*Atopobium vaginae*
*Firmicutes*		*Staphylococcus epidermidis*		
*Acinetobacter lwoffii*		*Fusobacterium* ssp.		
		*Enterococci*		
		*Bacteriodes* species		

## 5 Bacterial Infections Associated With Ovarian Cancer

Ovarian cancer is one of the most common deadly of gynecological cancers and risk of this cancer is about 1 in 78 women. According to American cancer society's statistics center, 22 530 new cases were estimated, which were 2.5% of all novel cancer cases, and 13 980 mortalities were estimated that 5% of all cancer deaths in 2019 (www.cancer.org). In addition, ovarian cancer is fifth causes of cancer‐related mortality among women and most commonly occurs in 63 years old or older and white women (www.cancer.org). Identified risk factors associated with this cancer are included hereditary factors such as Lynch syndrome (hereditary nonpolyposis colon cancer), alteration or mutation of *BRCA1* and *BRCA2* genes (tumor suppressor genes) and family history of either breast or ovarian cancer [47, 48]. The risk of ovarian cancer is assessed to be 40%–50% and 10%–25% for *BRCA1* and *BRCA2* mutation‐carriers, respectively [49]. The BARD protein interacts with p53 protein to regulate cell division and promote apoptosis or control cell death [50].

In addition, infertility, late menopause, hormone replacement therapy with androgens and estrogen, pelvic inflammatory disease (PID), endometriosis, polycystic ovary syndrome, lifestyle (obesity), and geography can be associated with an increased risk of ovarian cancer [51]. A study showed that breastfeeding and pregnancy frequency decrease ovarian cancer risk [52]. Several studies have been shown that bacterial infections are another important risk factor for the malignancy.

*Chlamydia trachomatis* is one of the most prevalent sexually transmitted microorganisms in the world [53]. Initial infection with *C. trachomatis* intracellular exhibits an asymptomatic period and may persist for years that reveals failure of the host immune responses [54]. The innate immune system initiates by the binding receptors named pattern of recognitions (PRRs) such as the nucleotide‐binding oligomerization domain proteins (NODs) and the Toll‐like receptors (TLRs) to pathogen‐associated molecular patterns (PAMPs), activating NF‐ĸB that NF‐ĸB attaches to nuclear DNA thus producing pro‐inflammatory cytokines [55]. These pro‐inflammatory cytokines are included IL‐6, IL‐8, IL‐10, IL‐12, and IFN‐γ. Macrophages and neutrophils that induce inflammatory responses, stimulate tissue damage and inhibit host cell apoptosis that can help ovarian carcinogenesis subsequent *Chlamydia* infections [52, 56]. It has been shown that 80% of OC cases were positive for *Chlamydia* infections [57]. HSP60 is synthesized as an immune protein during infection with *Chlamydia* that contains common antigenic epitopes and can lead to stimulate immune responses [56]. Binging of TLRs to HSP60 induces production of adhesion factors and the inflammatory chronic response [58]. HSP60 by inducing a series of reactions, by activation of caspase cascade, could inhibit apoptosis [59]. Tsai et al. have demonstrated that overexpression of HSP60 promotes tumorigenesis and metastasis [60]. The elevated levels of serum IgG antibody against HSP60 were found in ovarian cancer patients that is recognized as risk factor for the malignancy [61]. *C. trachomatis* can stimulate the production of reactive oxygen species (ROS) through the Mitochondrial Nod‐like Family Member NLRX1. ROS production is typically a cellular defense mechanism against invading microorganisms. However, *C. trachomatis* can override cellular defenses and utilize ROS to enhance its own growth by activating caspase‐1. The resulting elevated levels of ROS can cause to double‐stranded DNA damages in the infected cell. Moreover, *C. trachomatis* can diminish the DNA damage response (DDR) through inhibition of DDR proteins pATM and 53BP1 [62, 63]. In spite of the DNA damage, cells infected with Chlamydia continue to proliferate due to the activation of MAPK signaling and the expression of cyclin E [63]. Ultimately, *C. trachomatis can* trigger the proteasomal degradation of P53 through interaction with the phosphorylated ubiquitin ligase Murine Double Minute 2 (MDM2), resulting in inhibition of apoptosis. Combined, these mechanisms enhance bacterial survival, but they can also potentially lead to genomic instability, disruption of the cell cycle, and inhibition of apoptosis, all of which are key characteristics of cancer [63, 64, 65].

*Mycoplasma genitalium* is another sexually transmitted bacterium that causes urethritis, pelvic inflammatory disease (PID), cervicitis, vaginitis and infertility in females [66]. Banerjee et al. showed that 74% of the malignant ovarian cancer samples contained *M. genitalium* [67]. The another study was detected increased levels of serum *M. genitalium* IgG antibodies in ovarian cancer patients in comparison to controls [57]. *M. genitalium* produces toxins, which can lead to swelling of the fallopian tube epithelium cells that are the origin of ovarian cancer [68]. A study by Zhou et al. using 16S rRNA high‐throughput sequencing methods and normal distal fallopian tube transcriptome‐sequencing (RNA‐seq) analyses of ovarian cancer and tissues demonstrated that *Proteobacteria*, *Firmicutes*, and *Acinetobacter lwoffii* are associated with ovarian cancer [69].

## 6 Bacterial Infections Associated With Uterine/Endometrial Cancer

Uterine cancer is the most prevalent type of gynecological cancer and the fourth most prevalent cancer with about 61 880 new cases (7% of all cancer cases) with 12 160 deaths (4% of all tumor deaths) among the US women in 2019 (www.cancer.org). This cancer is more common in women between the ages of 55 and 74 and white women (www. cancer.org).

The 80% of the causative agents of endometrial cancer are associated with the environmental and host factors including increasing age, obesity, diabetes mellitus, late menopause, having no children, long‐term use of tamoxifen and excessive use of estrogen [70]. The remaining 20% of cases are related to Lynch syndrome, Cowden syndrome and family history of breast, ovarian and/or endometrial cancer [71]. Bacterial infections are also involved in the malignancy. The pathogenic bacteria could cause to boost via the vaginal or cervix into the upper genital tract and lead to PID, the uterus and endothelial dysfunction, thus promoting carcinogenesis [72]. Recent discoveries have challenged the traditional view of the uterus as a completely sterile environment [73]. New research suggests that the endometrium actually hosts a distinct community of microorganisms that can influence how tumors interact with surrounding tissues and affect cancer cell signaling [74]. In studies involving patients with endometrial cancer, researchers have consistently found that certain groups of bacteria are particularly prevalent. These include Bacteroidetes, such as *Bacteroides*, *Porphyromonas*, *Flavobacterium*, and *Prevotella*; Actinobacteria, with *Atopobium* being a notable member; Firmicutes, which includes *Anaerococcus*, *Dialister*, *Peptoniphilus*; and *Proteobacteria*, represented by *Pseudomonas* and *Acidovorax*. This highlights the diverse microbial life present in the endometrium and its potential impact on health [75, 76, 77].

## 7 Bacterial Infections Associated With Cervical Cancer

The carcinoma of the cervix is one of the most prevalent tumors in women. The malignancy is the fourth cause of cancer‐related deaths in women worldwide [78]. Squamous cell carcinoma and adenocarcinoma are the most important kinds of cervical cancers [79]. Continuous infection with HR‐HPV is an important factor in the promotion of cervical cancer [80]. Besides that, several risk factors such as smoking, multiple sexual partners, marrying before age 18 years old, multiple childbirths, and bacterial infections have been known for the malignancy [81].

There is a microbial balance in the vagina. The most common microorganisms in the intact vagina are *Lactobacillus* spp., which are also involved in the defense of the reproductive system. The disruption in the balance of vaginal flora during physiological conditions results in numerous gynecological diseases, such as colitis, high‐grade cervical intraepithelial neoplasia (CIN) and cervical cancer [82]. Reduction of the *Lactobacillus* spp. population results in overgrowing of anaerobic infections [83]. Up to now, the link among vaginal bacteria and cervical cancer has not been identified; but several researches have reported that the frequency of various bacteria such as *Gardnerella vaginalis* , *Prevotella bivia* , *Mycoplasma genitalium* , *Staphylococcus epidermidis* , *Enterococcus* spp., *Escherichia coli* , *Fusobacterium* spp., and *Bacteroides* spp. is different in the cancerous women in comparison to healthy women [84, 85, 86]. These bacteria promote tumorogenesis through the activation of host immunity and production of various metabolites. Anaerobe bacteria release high levels of polyamines and organic acids including acetic and succinic acids, which are toxic to the cells. The evaluated levels of nitrosamines lead to DNA breakdown and change cytokine releasing that influences the immune responses to the HPV. So, the anaerobic bacteria promote the risk of HPV infections and cervical cancer [87, 88]. Also, the bacteria release numerous immunomodulatory factors including proteases, sialidases, and succinate [89]. In addition, inflammatory‐inducing substances such as lipoteichoic acid, peptidoglycans and LPS can result in the release of proinflammatory cytokines such as IL‐6 and IL‐8 [88]. It has been reported that the cytokines promote angiogenesis and carcinogenesis of solid cancers [90]. In addition, the vaginal bacteria activate TGFβ through activation of TLR. TGFβ promotes the expression of integrins and progresses the invasion of solid tumors [91].

## 8 Bacterial Infections Associated With Vulvar Cancer

Vulvar cancer is a rare type of cancer that accounts for approximately 5% of gynecological cancer. Vulvar cancer is raised from the tissues of the vulva and usually affects postmenopausal women. There were 6170 new cases of vulvar cancer (0.3% of all new cancer cases), with 1280 deaths a year (0.2% of all cancer deaths) in the United States in 2019. This cancer is often observed in women after the age of 45 and from lower social classes [92].

The increasing age, smoking cigarettes, having chronic skin conditions and inflammation, history of vulvar or cervical intraepithelial neoplasia, weakened immune system (HIV infection) and Human papilloma virus (HPV) infection increase the risks of vulvar cancer [93]. As with the other gynecological cancers, bacterial infections are involved in the malignancy. Boutas et al. [94] reported that *Bacteroides fragilis* ( *B. fragilis* ) was found in a 78‐year‐old woman with vulvar cancer. *B. fragilis* can promote carcinogenesis via production of fragilysin and metalloproteinase, which lead to the stimulation of synthesis of cyclin D and c‐Myc proteins (as transcription factors) by releasing β‐catenin [95]. In addition, fragilysin triggers activation of STAT3 transcription factors and NF‐κB that activates the synthesis of proinflammatory cytokines with oncogenic effects [95, 96].

In addition, there is an association between *C. trachomatis* infection and vulvar cancer [97]. In addition, *C. trachomatis* antigen was detected in 20% of patients with vulvar cancer, and antibodies of IgM and IgG *Chlamydia* were found in 13.3% and 50% of these patients [98].

## 9 Bacterial Infections Associated With Vaginal Cancer

Vaginal cancer is one of the rarest of gynecological cancers and is only 2% of all gynecological cancers with about 1 of every 1100 women. It was estimated that 5170 new cases and 1430 deaths in the United States in 2019 (www.cancer.org). The risk of vaginal cancer is associated with women 70 years old or older in more than half of the cases, prenatal exposure to diethylstilbestrol, smoking, a previous history of cervical cancer, and viral infections such as human papillomavirus (HPV 16 and HPV 18) and human immunodeficiency virus (HIV) [99]. In addition, bacterial infections may be involved in the pathogenesis of vaginal cancer. The most common causes of bacteria in vaginal cancer are *Lactobacillus* spp. including *L. iners* , *L. crispatus* , *L. jensenii* , and *L. gasseri* , with *L. iners*, and *L. crispatus* being the dominant species among them [100]. *Lactobacillus* spp. through the induction of cytokine production including TNF‐α, IFN‐γ, IL‐12, IL‐18, Nod2, and TLR2 activate innate immune responses to protect the genital tract host [101]. On the other hand, *Lactobacillus* spp. through lactic acid production provide a pH > 4.5, which prevents the invasion of pathogenic bacteria in the vaginal environment [102]. *Mycoplasma spp*. is a commensal bacterium of humans that switch into a pathogenic state by altered host immunity or microbial environment, causing vaginal cancer [103].

Alteration of the vaginal flora, a decrease in the proportion of *Lactobacillus* spp., can lead to increase incidence of bacterial vaginosis such as *Gardnerella vaginalis* , *Atopobium vaginae* , *Mycoplasma* spp., *Clostridiales*, *Prevotella* spp., and *Megasphaera* spp. [104]. The vaginal *Lactobacillus* spp. seem to be protective, and a disruption of them can cause reduced immune protection and damage the epithelial lining of reproductive tissues, as well as, replacement of bacterial vaginosis, thereby promoting carcinogenesis [105].

The important known bacteria, which promote inflammation in vagina are *A. vaginae* , *G. vaginalis* , and *Mycoplasma hominis* . *A. vaginae* induces pro‐inflammatory cytokines IL‐6, IL‐8, and the antimicrobial peptide b‐defensin 4 via the binding TLR1, −2 and −6 to NF‐kB signaling from vaginal epithelial cells [106]. *G. vaginalis* induces IL‐6 and IL‐8 and *Mycoplasma hominis* induces TNFα, which are involved in inflammation and may be in cancer development [107].

## 10 Conclusion

Gynecological cancers are one of the most common cancers in women that affect their reproductive organs. The role of bacterial infection as an external factor has been established in several cancers, as well as gynecological cancers. However, the mechanisms of carcinogenesis of bacteria are unclear. Women's reproductive organs have a dynamic and relative microbial balance. The disruption in the balance of the microbiome could result in numerous gynecological diseases and gynecological cancers. Among the different bacterial infections, infections caused by *Chlamydia*, *Mycoplasma*, and *Bacteriodes* species are important infections associated with the malignancies. Evaluation of the microbiome in the reproductive organs of gynecological cancer patients and studies on the prevention and control of the infections in the patients could be useful in verifying the pathogenesis of the diseases and also the identification of suitable therapeutic strategies.

## Author Contributions

**Robab Azargun:** methodology, writing – original draft. **Maryam Azargoon:** methodology, writing – review and editing. **Zahra Asefy:** investigation, writing – review and editing. **Mina Yekani:** software, writing – review and editing. **Vahideh Tarhriz:** investigation, writing – review and editing. **Fatemeh Yeganeh:** methodology, writing – original draft. **Mohammad Yousef Memar:** supervision, writing – review and editing. **Shirin Eyvazi:** validation, writing – review and editing.

## Funding

The authors have nothing to report.

## Ethics Statement

The authors have nothing to report.

## Conflicts of Interest

The authors declare no conflicts of interest.

## Supporting information


**Figure S1:** An overview of the literature search strategy.

## Data Availability

Data sharing not applicable to this article as no datasets were generated or analyzed during the current study.

## References

[1] G. Cocomazzi , L. Del Pup , V. Contu , et al., “Gynecological Cancers and Microbiota Dynamics: Insights Into Pathogenesis and Therapy,” International Journal of Molecular Sciences 25, no. 4 (2024): 2237. 38396914 10.3390/ijms25042237 PMC10889201

[2] J. Chalif , H. Wang , D. Spakowicz , et al., “The Microbiome and Gynecologic Cancer: Cellular Mechanisms and Clinical Applications,” International Journal of Gynecological Cancer 34, no. 2 (2024): 317–327. 38088183 10.1136/ijgc-2023-004894

[3] J. Ferlay , I. Soerjomataram , R. Dikshit , et al., “Cancer Incidence and Mortality Worldwide: Sources, Methods and Major Patterns in GLOBOCAN 2012,” International Journal of Cancer 136, no. 5 (2015): E359–E386. 25220842 10.1002/ijc.29210

[4] G. C. Jayson , E. C. Kohn , H. C. Kitchener , and J. A. Ledermann , “Ovarian Cancer,” Lancet 384, no. 9951 (2014): 1376–1388. 24767708 10.1016/S0140-6736(13)62146-7

[5] L. M. Randall and B. Pothuri , “The Genetic Prediction of Risk for Gynecologic Cancers,” Gynecologic Oncology 141, no. 1 (2016): 10–16. 27016223 10.1016/j.ygyno.2016.03.007

[6] S. Ahmadi , M. Taghizadieh , E. Mehdizadehfar , et al., “Gut Microbiota in Neurological Diseases: Melatonin Plays an Important Regulatory Role,” Biomedicine & Pharmacotherapy 174 (2024): 116487. 38518598 10.1016/j.biopha.2024.116487

[7] E. Nabizadeh , M. Y. Memar , H. Hamishehkar , et al., “Short‐Chain Fatty Acids Profile in Patients With SARS‐CoV‐2: A Case‐Control Study,” Health Science Reports 6, no. 7 (2023): e1411. 37425235 10.1002/hsr2.1411 PMC10323717

[8] M. Han , N. Wang , W. Han , M. Ban , T. Sun , and J. Xu , “Vaginal and Tumor Microbiomes in Gynecological Cancer,” Oncology Letters 25, no. 4 (2023): 153. 36936020 10.3892/ol.2023.13739 PMC10018329

[9] Z. Wang , L. Zhang , X. Liu , and L. Xu , “The Role of Reproductive Tract Microbiota in Gynecological Health and Diseases,” Journal of Reproductive Immunology 167 (2025): 104418. 39700680 10.1016/j.jri.2024.104418

[10] S. Nicolò , A. Antonelli , M. Tanturli , et al., “Bacterial Species From Vaginal Microbiota Differently Affect the Production of the E6 and E7 Oncoproteins and of p53 and p‐Rb Oncosuppressors in HPV16‐Infected Cells,” International Journal of Molecular Sciences 24, no. 8 (2023): 7173. 37108333 10.3390/ijms24087173 PMC10138431

[11] H. Elkafas , M. Walls , A. Al‐Hendy , and N. Ismail , “Gut and Genital Tract Microbiomes: Dysbiosis and Link to Gynecological Disorders,” Frontiers in Cellular and Infection Microbiology 12 (2022): 1059825. 36590579 10.3389/fcimb.2022.1059825 PMC9800796

[12] J. Shen , H. Sun , J. Chu , X. Gong , and X. Liu , “Cervicovaginal Microbiota: A Promising Direction for Prevention and Treatment in Cervical Cancer,” Infectious Agents and Cancer 19, no. 1 (2024): 13. 38641803 10.1186/s13027-024-00573-8 PMC11027553

[13] J. S. Townsend , M. Puckett , C. A. Gelb , M. Whiteside , J. Thorsness , and S. L. Stewart , “Improving Knowledge and Awareness of HPV‐Associated Gynecologic Cancers: Results From the National Comprehensive Cancer Control Program/Inside Knowledge Collaboration,” 2018. 10.1089/jwh.2018.7289 PMC6169312 30129896

[14] A. J. B. Smith , S. Varma , A. F. Rositch , and K. L. Levinson , “Gynecologic Cancer in HIV‐Positive Women: A Systematic Review and Meta‐Analysis,” American Journal of Obstetrics and Gynecology 221 (2019): 194–207.e5. 30771344 10.1016/j.ajog.2019.02.022

[15] Q. Zou , Y. Wu , S. Zhang , et al., “Escherichia Coli and HPV16 Coinfection May Contribute to the Development of Cervical Cancer,” Virulence 15, no. 1 (2024): 2319962. 38380669 10.1080/21505594.2024.2319962 PMC10883084

[16] S.‐Y. Choi and J.‐H. Choi , “Ovarian Cancer and the Microbiome: Connecting the Dots for Early Diagnosis and Therapeutic Innovations—A Review,” Medicina (Kaunas, Lithuania) 60, no. 3 (2024): 516. 38541242 10.3390/medicina60030516 PMC10972291

[17] X. Wang , Y. Zheng , X. Chen , et al., “2bRAD‐M Reveals the Difference in Microbial Distribution Between Cancerous and Benign Ovarian Tissues,” Frontiers in Microbiology 14 (2023): 1231354. 37692387 10.3389/fmicb.2023.1231354 PMC10484612

[18] P. Correa , “Bacterial Infections as a Cause of Cancer,” Journal of the National Cancer Institute 95, no. 7 (2003): E3–E. 12671026 10.1093/jnci/95.7.e3

[19] P. Gholizadeh , M. Mahallei , A. Pormohammad , et al., “Microbial Balance in the Intestinal Microbiota and Its Association With Diabetes, Obesity and Allergic Disease,” Microbial Pathogenesis 127 (2019): 48–55. 30503960 10.1016/j.micpath.2018.11.031

[20] A. Clark , “The Hygiene Hypothesis Revisited: Autoimmune Diseases, Intestinal Microbiota and Vitamin D's Role,” 2016.

[21] A. V. Hartstra , K. E. Bouter , F. Bäckhed , and M. Nieuwdorp , “Insights Into the Role of the Microbiome in Obesity and Type 2 Diabetes,” Diabetes Care 38, no. 1 (2015): 159–165. 25538312 10.2337/dc14-0769

[22] S. Kapil , A. Duseja , B. K. Sharma , et al., “Small Intestinal Bacterial Overgrowth and Toll‐Like Receptor Signaling in Patients With Non‐Alcoholic Fatty Liver Disease,” Journal of Gastroenterology and Hepatology 31, no. 1 (2016): 213–221. 26212089 10.1111/jgh.13058

[23] F. Pistollato , S. Sumalla Cano , I. Elio , M. Masias Vergara , F. Giampieri , and M. Battino , “Role of Gut Microbiota and Nutrients in Amyloid Formation and Pathogenesis of Alzheimer Disease,” Nutrition Reviews 74, no. 10 (2016): 624–634. 27634977 10.1093/nutrit/nuw023

[24] P. Gholizadeh , H. Eslami , and H. S. Kafil , “Carcinogenesis Mechanisms of *Fusobacterium nucleatum* ,” Biomedicine & Pharmacotherapy 89 (2017): 918–925. 28292019 10.1016/j.biopha.2017.02.102

[25] P. Gholizadeh , H. Eslami , M. Yousefi , M. Asgharzadeh , M. Aghazadeh , and H. S. Kafil , “Role of Oral Microbiome on Oral Cancers, a Review,” Biomedicine & Pharmacotherapy 84 (2016): 552–558. 27693964 10.1016/j.biopha.2016.09.082

[26] A. Pormohammad , N. Mohtavinejad , P. Gholizadeh , et al., “Global Estimate of Gastric Cancer in *Helicobacter pylori*–Infected Population: A Systematic Review and Meta‐Analysis,” Journal of Cellular Physiology 234, no. 2 (2019): 1208–1218. 30132888 10.1002/jcp.27114

[27] S. Eyvazi , M. A. Vostakolaei , A. Dilmaghani , et al., “The Oncogenic Roles of Bacterial Infections in Development of Cancer,” Microbial Pathogenesis 141 (2020): 104019. 32006638 10.1016/j.micpath.2020.104019

[28] M. H. Vala , S. Eyvazi , H. Goudarzi , H. R. Sarie , and M. Gholami , “Evaluation of Clarithromycin Resistance Among Iranian *Helicobacter pylori* Isolates by E‐Test and Real‐Time Polymerase Chain Reaction Methods,” Jundishapur Journal of Microbiology 9, no. 5 (2016): e39336. 10.5812/jjm.29839 PMC4976621 27540451

[29] L. Mughini‐Gras , M. Schaapveld , J. Kramers , et al., “Increased Colon Cancer Risk After Severe Salmonella Infection,” PLoS One 13, no. 1 (2018): e0189721. 29342165 10.1371/journal.pone.0189721 PMC5771566

[30] L. Vannucci , R. Stepankova , H. Kozakova , A. Fiserova , P. Rossmann , and H. Tlaskalova‐Hogenova , “Colorectal Carcinogenesis in Germ‐Free and Conventionally Reared Rats: Different Intestinal Environments Affect the Systemic Immunity,” International Journal of Oncology 32, no. 3 (2008): 609–617. 18292938

[31] D. H. Dapito , A. Mencin , G.‐Y. Gwak , et al., “Promotion of Hepatocellular Carcinoma by the Intestinal Microbiota and TLR4,” Cancer Cell 21, no. 4 (2012): 504–516. 22516259 10.1016/j.ccr.2012.02.007 PMC3332000

[32] D. van Elsland and J. Neefjes , “Bacterial Infections and Cancer,” EMBO Reports 19, no. 11 (2018): e46632. 30348892 10.15252/embr.201846632 PMC6216254

[33] M. R. Rubinstein , X. Wang , W. Liu , Y. Hao , G. Cai , and Y. W. Han , “ *Fusobacterium Nucleatum* Promotes Colorectal Carcinogenesis by Modulating E‐Cadherin/β‐Catenin Signaling via Its FadA Adhesin,” Cell Host & Microbe 14, no. 2 (2013): 195–206. 23954158 10.1016/j.chom.2013.07.012 PMC3770529

[34] T. Wiedemann , S. Hofbaur , N. Tegtmeyer , et al., “ *Helicobacter pylori* CagL Dependent Induction of Gastrin Expression via a Novel αvβ5‐Integrin–Integrin Linked Kinase Signalling Complex,” Gut 61, no. 7 (2012): 986–996. 22287591 10.1136/gutjnl-2011-300525

[35] E. Talluri , L. Pancotto , P. Ruggiero , M. Scarselli , and E. Balducci , “CagL From *Helicobacter pylori* Has ADP‐Ribosylation Activity and Exerts Partial Protective Efficacy in Mice,” Archives of Biochemistry and Biophysics 635 (2017): 102–109. 29097311 10.1016/j.abb.2017.10.019

[36] X. Bao and J. Wu , “Natural Anti‐Adhesive Components Against Pathogenic Bacterial Adhesion and Infection in Gastrointestinal Tract: Case Studies of *Helicobacter pylori*, *Salmonella enterica*, Clostridium Difficile, and Diarrheagenic *Escherichia coli* ,” Critical Reviews in Food Science and Nutrition 65 (2024): 1–46. 10.1080/10408398.2024.2436139 39666022

[37] H. Ogawa , A. Iwamoto , T. Tanahashi , et al., “Genetic Variants of *Helicobacter pylori* Type IV Secretion System Components CagL and CagI and Their Association With Clinical Outcomes,” Gut Pathogens 9, no. 1 (2017): 21. 28439300 10.1186/s13099-017-0165-1 PMC5399799

[38] M. R. Aliramaei , M. R. Khorasgani , M. R. Rahmani , S. H. Z. Esfahani , and R. Emamzadeh , “Expression of *Helicobacter pylori* CagL Gene in *Lactococcus lactis* MG1363 and Evaluation of Its Immunogenicity as an Oral Vaccine in Mice,” Microbial Pathogenesis 142 (2020): 103926. 31838174 10.1016/j.micpath.2019.103926

[39] N. Ishijima , M. Suzuki , H. Ashida , et al., “BabA‐Mediated Adherence Is a Potentiator of the *Helicobacter pylori* Type IV Secretion System Activity,” Journal of Biological Chemistry 286, no. 28 (2011): 25256–25264. 21596743 10.1074/jbc.M111.233601 PMC3137096

[40] D. Yesudhas , V. Gosu , M. A. Anwar , and S. Choi , “Multiple Roles of Toll‐Like Receptor 4 in Colorectal Cancer,” Frontiers in Immunology 5 (2014): 334. 25076949 10.3389/fimmu.2014.00334 PMC4097957

[41] E. G. Di Domenico , I. Cavallo , M. Pontone , L. Toma , and F. Ensoli , “Biofilm Producing *Salmonella typhi* : Chronic Colonization and Development of Gallbladder Cancer,” International Journal of Molecular Sciences 18, no. 9 (2017): 1887. 28858232 10.3390/ijms18091887 PMC5618536

[42] L. Guerra , H. S. Carr , A. Richter‐Dahlfors , et al., “A Bacterial Cytotoxin Identifies the RhoA Exchange Factor Net1 as a Key Effector in the Response to DNA Damage,” PLoS One 3, no. 5 (2008): e2254. 18509476 10.1371/journal.pone.0002254 PMC2386254

[43] J. Putze , C. Hennequin , J.‐P. Nougayrède , et al., “Genetic Structure and Distribution of the Colibactin Genomic Island Among Members of the Family Enterobacteriaceae,” Infection and Immunity 77, no. 11 (2009): 4696–4703. 19720753 10.1128/IAI.00522-09 PMC2772509

[44] X. Yong , B. Tang , B.‐S. Li , et al., “ *Helicobacter pylori* Virulence Factor CagA Promotes Tumorigenesis of Gastric Cancer via Multiple Signaling Pathways,” Cell Communication and Signaling 13, no. 1 (2015): 30. 26160167 10.1186/s12964-015-0111-0 PMC4702319

[45] T. Scanu , R. M. Spaapen , J. M. Bakker , et al., “Salmonella Manipulation of Host Signaling Pathways Provokes Cellular Transformation Associated With Gallbladder Carcinoma,” Cell Host & Microbe 17, no. 6 (2015): 763–774. 26028364 10.1016/j.chom.2015.05.002

[46] R. Lu , M. Bosland , Y. Xia , Y.‐g. Zhang , I. Kato , and J. Sun , “Presence of Salmonella AvrA in Colorectal Tumor and Its Precursor Lesions in Mouse Intestine and Human Specimens,” Oncotarget 8, no. 33 (2017): 55104. 28903406 10.18632/oncotarget.19052 PMC5589645

[47] M. Søgaard , S. Krüger Kjær , and S. Gayther , “Ovarian Cancer and Genetic Susceptibility in Relation to the BRCA1 and BRCA2 Genes. Occurrence, Clinical Importance and Intervention,” Acta Obstetricia et Gynecologica Scandinavica 85, no. 1 (2006): 93–105. 16521688 10.1080/00016340500324621

[48] M. D. Schwartz , B. N. Peshkin , K. P. Tercyak , K. L. Taylor , and H. Valdimarsdottir , “Decision Making and Decision Support for Hereditary Breast‐Ovarian Cancer Susceptibility,” Health Psychology 24, no. 4S (2005): S78–S84. 16045423 10.1037/0278-6133.24.4.S78

[49] J. O. Schorge , S. C. Modesitt , R. L. Coleman , et al., “SGO White Paper on Ovarian Cancer: Etiology, Screening and Surveillance,” Gynecologic Oncology 119, no. 1 (2010): 7–17. 20692025 10.1016/j.ygyno.2010.06.003

[50] A. Desai , J. Xu , K. Aysola , et al., “Epithelial Ovarian Cancer: An Overview,” World Journal of Translational Medicine 3, no. 1 (2014): 1–8. 25525571 10.5528/wjtm.v3.i1.1 PMC4267287

[51] J. Hunn and G. C. Rodriguez , “Ovarian Cancer: Etiology, Risk Factors, and Epidemiology,” Clinical Obstetrics and Gynecology 55, no. 1 (2012): 3–23. 22343225 10.1097/GRF.0b013e31824b4611

[52] X. Xie , M. Yang , Y. Ding , and J. Chen , “Microbial Infection, Inflammation and Epithelial Ovarian Cancer,” Oncology Letters 14, no. 2 (2017): 1911–1919. 28789426 10.3892/ol.2017.6388 PMC5529868

[53] Organization WH , Global Prevalence and Incidence of Selected Curable Sexually Transmitted Infections: Overview and Estimates (World Health Organization, 2001).

[54] M. Molano , C. J. Meijer , E. Weiderpass , et al., “The Natural Course of *Chlamydia trachomatis* Infection in Asymptomatic Colombian Women: A 5‐Year Follow‐Up Study,” Journal of Infectious Diseases 191, no. 6 (2005): 907–916. 15717266 10.1086/428287

[55] H. Sellami , N. Said‐Sadier , A. Znazen , R. Gdoura , D. M. Ojcius , and A. Hammami , “ *Chlamydia trachomatis* Infection Increases the Expression of Inflammatory Tumorigenic Cytokines and Chemokines as Well as Components of the Toll‐Like Receptor and NF‐κB Pathways in Human Prostate Epithelial Cells,” Molecular and Cellular Probes 28, no. 4 (2014): 147–154. 24613856 10.1016/j.mcp.2014.01.006

[56] M. T. Mascellino , P. Boccia , and A. Oliva , “Immunopathogenesis in *Chlamydia trachomatis* Infected Women,” ISRN Obstetrics and Gynecology 2011 (2011): 1–9. 10.5402/2011/436936 PMC3236400 22191045

[57] A. Idahl , E. Lundin , M. Jurstrand , et al., “Chlamydia Trachomatis and *Mycoplasma genitalium* Plasma Antibodies in Relation to Epithelial Ovarian Tumors,” Infectious Diseases in Obstetrics and Gynecology 2011 (2011): 1–10. 10.1155/2011/824627 PMC3147007 21811380

[58] R. S. Stephens , “The Cellular Paradigm of Chlamydial Pathogenesis,” Trends in Microbiology 11, no. 1 (2003): 44–51. 12526854 10.1016/s0966-842x(02)00011-2

[59] V. Di Felice , S. David , F. Cappello , F. Farina , and G. Zummo , “Is Chlamydial Heat Shock Protein 60 a Risk Factor for Oncogenesis?,” Cellular and Molecular Life Sciences: CMLS 62, no. 1 (2005): 4–9. 15619002 10.1007/s00018-004-4367-6 PMC11924578

[60] Y.‐P. Tsai , M.‐H. Yang , C.‐H. Huang , et al., “Interaction Between HSP60 and β‐Catenin Promotes Metastasis,” Carcinogenesis 30, no. 6 (2009): 1049–1057. 19369584 10.1093/carcin/bgp087

[61] P. Bodzek , R. Partyka , and A. Damasiewicz‐Bodzek , “Antibodies Against Hsp60 and Hsp65 in the Sera of Women With Ovarian Cancer,” Journal of Ovarian Research 7, no. 1 (2014): 30. 24618330 10.1186/1757-2215-7-30 PMC3984705

[62] A. A. Abdul‐Sater , N. Saïd‐Sadier , V. M. Lam , et al., “Enhancement of Reactive Oxygen Species Production and Chlamydial Infection by the Mitochondrial Nod‐Like Family Member NLRX1,” Journal of Biological Chemistry 285, no. 53 (2010): 41637–41645. 20959452 10.1074/jbc.M110.137885 PMC3009891

[63] C. Chumduri , R. K. Gurumurthy , P. K. Zadora , Y. Mi , and T. F. Meyer , “Chlamydia Infection Promotes Host DNA Damage and Proliferation but Impairs the DNA Damage Response,” Cell Host & Microbe 13, no. 6 (2013): 746–758. 23768498 10.1016/j.chom.2013.05.010

[64] E. González , M. Rother , M. C. Kerr , et al., “Chlamydia Infection Depends on a Functional MDM2‐p53 Axis,” Nature Communications 5, no. 1 (2014): 5201. 10.1038/ncomms6201 PMC4243245 25392082

[65] D. Hanahan and R. A. Weinberg , “Hallmarks of Cancer: The Next Generation,” Cell 144, no. 5 (2011): 646–674. 21376230 10.1016/j.cell.2011.02.013

[66] D. Taylor‐Robinson and J. S. Jensen , “ *Mycoplasma genitalium* : From Chrysalis to Multicolored Butterfly,” Clinical Microbiology Reviews 24, no. 3 (2011): 498–514. 21734246 10.1128/CMR.00006-11 PMC3131060

[67] S. Banerjee , T. Tian , Z. Wei , et al., “The Ovarian Cancer Oncobiome,” Oncotarget 8, no. 22 (2017): 36225. 28410234 10.18632/oncotarget.16717 PMC5482651

[68] C. P. Crum , F. D. McKeon , and W. Xian , “The Oviduct and Ovarian Cancer: Causality, Clinical Implications and “Targeted Prevention”,” Clinical Obstetrics and Gynecology 55, no. 1 (2012): 24–35. 22343226 10.1097/GRF.0b013e31824b1725 PMC3319355

[69] B. Zhou , C. Sun , J. Huang , et al., “The Biodiversity Composition of Microbiome in Ovarian Carcinoma Patients,” Scientific Reports 9, no. 1 (2019): 1691. 30737418 10.1038/s41598-018-38031-2 PMC6368644

[70] F. Amant , P. Moerman , P. Neven , D. Timmerman , E. Van Limbergen , and I. Vergote , “Endometrial Cancer,” Lancet 366, no. 9484 (2005): 491–505. 16084259 10.1016/S0140-6736(05)67063-8

[71] M. R. Walther‐António , J. Chen , F. Multinu , et al., “Potential Contribution of the Uterine Microbiome in the Development of Endometrial Cancer,” Genome Medicine 8, no. 1 (2016): 122. 27884207 10.1186/s13073-016-0368-y PMC5123330

[72] R. F. Schwabe and C. Jobin , “The Microbiome and Cancer,” Nature Reviews Cancer 13, no. 11 (2013): 800. 24132111 10.1038/nrc3610 PMC3986062

[73] L. C. Giudice , “Challenging Dogma: The Endometrium Has a Microbiome With Functional Consequences!,” American Journal of Obstetrics and Gynecology 215, no. 6 (2016): 682–683. 27725136 10.1016/j.ajog.2016.09.085

[74] G. Ventolini , P. Vieira‐Baptista , F. De Seta , H. Verstraelen , R. Lonnee‐Hoffmann , and A. Lev‐Sagie , “The Vaginal Microbiome: IV. The Role of Vaginal Microbiome in Reproduction and in Gynecologic Cancers,” Journal of Lower Genital Tract Disease 26, no. 1 (2022): 93–98. 34928259 10.1097/LGT.0000000000000646 PMC8719507

[75] G. Stabile , A. Doria , M. Bruno , et al., “The Role of the Endometrial Microbiota in Endometrial Cancer: A Systematic Review of the Literature,” Journal of Clinical Medicine 13, no. 23 (2024): 7135. 39685594 10.3390/jcm13237135 PMC11642298

[76] G. M. Hawkins , W. C. Burkett , A. N. McCoy , et al., “Differences in the Microbial Profiles of Early Stage Endometrial Cancers Between Black and White Women,” Gynecologic Oncology 165, no. 2 (2022): 248–256. 35277280 10.1016/j.ygyno.2022.02.021 PMC9093563

[77] L. Wang , J. Yang , H. Su , L. Shi , B. Chen , and S. Zhang , “Endometrial Microbiota From Endometrial Cancer and Paired Pericancer Tissues in Postmenopausal Women: Differences and Clinical Relevance,” Menopause 29, no. 10 (2022): 1168–1175. 36150116 10.1097/GME.0000000000002053 PMC9512232

[78] O. Ginsburg , F. Bray , M. P. Coleman , et al., “The Global Burden of Women's Cancers: A Grand Challenge in Global Health,” Lancet 389, no. 10071 (2017): 847–860. 27814965 10.1016/S0140-6736(16)31392-7 PMC6191029

[79] C. J. Meijer and R. D. Steenbergen , “Novel Molecular Subtypes of Cervical Cancer—Potential Clinical Consequences,” Nature Reviews Clinical Oncology 14, no. 7 (2017): 397–398. 10.1038/nrclinonc.2017.52 28397825

[80] G. O. Chong , Y. H. Lee , H. S. Han , et al., “Prognostic Value of Pre‐Treatment Human Papilloma Virus DNA Status in Cervical Cancer,” Gynecologic Oncology 148, no. 1 (2018): 97–102. 29153540 10.1016/j.ygyno.2017.11.003

[81] D. Dodd and C. Boyd , “Populations at Risk for Cervical Cancer,” 2018.

[82] E. Amabebe and D. O. Anumba , “The Vaginal Microenvironment: The Physiologic Role of Lactobacilli,” Frontiers in Medicine 5 (2018): 181. 29951482 10.3389/fmed.2018.00181 PMC6008313

[83] M. Di Paola , C. Sani , A. M. Clemente , et al., “Characterization of Cervico‐Vaginal Microbiota in Women Developing Persistent High‐Risk Human Papillomavirus Infection,” Scientific Reports 7, no. 1 (2017): 10200. 28860468 10.1038/s41598-017-09842-6 PMC5579045

[84] H. Mikamo , K. Izumi , K. Ito , K. Watanabe , K. Ueno , and T. Tamaya , “Internal Bacterial Flora of Solid Uterine Cervical Cancer,” Kansenshōgaku Zasshi 67, no. 11 (1993): 1057–1061. 8270797 10.11150/kansenshogakuzasshi1970.67.1057

[85] A. Audirac‐Chalifour , K. Torres‐Poveda , M. Bahena‐Roman , et al., “Cervical Microbiome and Cytokine Profile at Various Stages of Cervical Cancer: A Pilot Study,” PLoS One 11, no. 4 (2016): e0153274. 27115350 10.1371/journal.pone.0153274 PMC4846060

[86] H. Mikamo , Y. Sato , Y. Hayasaki , et al., “Intravaginal Bacterial Flora in Patients With Uterine Cervical Cancer. High Incidence of Detection of *Gardnerella vaginalis* ,” Journal of Infection and Chemotherapy 5, no. 2 (1999): 82–85. 11810495 10.1007/s101560050013

[87] N. Pavic , “Is There a Local Production of Nitrosamines by the Vaginal Microflora in Anaerobic Vaginosis/Trichomoniasis?,” Medical Hypotheses 15, no. 4 (1984): 433–436. 6396500 10.1016/0306-9877(84)90159-2

[88] B. M. Biswal , K. K. B. Singh , M. B. Ismail , M. I. B. A. Jalal , and E. I. S. B. E. Safruddin , “Current Concept of Bacterial Vaginosis in Cervical Cancer,” Journal of Clinical Gynecology and Obstetrics 3, no. 1 (2014): 1–7.

[89] P. Sudhakara , I. Sellamuthu , and A. W. Aruni , “Bacterial Sialoglycosidases in Virulence and Pathogenesis,” Pathogens 8, no. 1 (2019): 39. 30909660 10.3390/pathogens8010039 PMC6471121

[90] Q. Huang , L. Duan , X. Qian , et al., “IL‐17 Promotes Angiogenic Factors IL‐6, IL‐8, and Vegf Production via Stat1 in Lung Adenocarcinoma,” Scientific Reports 6 (2016): 36551. 27819281 10.1038/srep36551 PMC5098156

[91] J. S. Desgrosellier and D. A. Cheresh , “Integrins in Cancer: Biological Implications and Therapeutic Opportunities,” Nature Reviews. Cancer 10, no. 1 (2010): 9–22. 20029421 10.1038/nrc2748 PMC4383089

[92] M. N. De Koning , W. G. Quint , and E. C. Pirog , “Prevalence of Mucosal and Cutaneous Human Papillomaviruses in Different Histologic Subtypes of Vulvar Carcinoma,” Modern Pathology 21, no. 3 (2008): 334. 18192968 10.1038/modpathol.3801009

[93] B. S. Madsen , H. L. Jensen , A. J. van den Brule , J. Wohlfahrt , and M. Frisch , “Risk Factors for Invasive Squamous Cell Carcinoma of the Vulva and Vagina—Population‐Based Case–Control Study in Denmark,” International Journal of Cancer 122, no. 12 (2008): 2827–2834. 18348142 10.1002/ijc.23446

[94] I. Boutas , C. Sofoudis , E. Kalampokas , C. Anastasopoulos , T. Kalampokas , and N. Salakos , “Verrucous Carcinoma of the Vulva: A Case Report,” Case Reports in Obstetrics and Gynecology 2013 (2013): 932712. 23401817 10.1155/2013/932712 PMC3562618

[95] T. Zykova , O. I. Kit , G. A. Nerodo , et al., On Bacteroides Fragilis Participation in Vulvar Carcinogenesis (American Society of Clinical Oncology, 2017).

[96] M. Yekani , H. B. Baghi , B. Naghili , S. Z. Vahed , J. Sóki , and M. Y. Memar , “To Resist and Persist: Important Factors in the Pathogenesis of *Bacteroides fragilis* ,” Microbial Pathogenesis 149 (2020): 104506. 32950639 10.1016/j.micpath.2020.104506

[97] A. Kwaśniewska , E. Korobowicz , J. Visconti , M. Zdunek , M. Szymańiski , and A. Goździcka‐Józefiak , “Chlamydia Trachomatis and Herpes Simplex Virus 2 Infection in Vulvar Intraepithelial Neoplasia Associated With Human Papillomavirus,” European Journal of Gynaecological Oncology 27, no. 4 (2006): 405–408. 17009637

[98] A. Olejek , I. Kozak‐Darmas , S. Kellas‐Sleczka , et al., “ *Chlamydia trachomatis* Infection in Women With Lichen Sclerosus Vulvae and Vulvar Cancer,” Neuro Endocrinology Letters 30, no. 5 (2009): 671–674. 20035265

[99] M. J. Merino , “Vaginal Cancer: The Role of Infectious and Environmental Factors,” American Journal of Obstetrics and Gynecology 165, no. 4 (1991): 1255–1262. 1659200 10.1016/s0002-9378(12)90738-3

[100] J. Ravel , P. Gajer , Z. Abdo , et al., “Vaginal Microbiome of Reproductive‐Age Women,” National Academy of Sciences of the United States of America 108, no. Supplement 1 (2011): 4680–4687. 10.1073/pnas.1002611107 PMC3063603 20534435

[101] Y.‐G. Kim , T. Ohta , T. Takahashi , et al., “Probiotic *Lactobacillus casei* Activates Innate Immunity via NF‐κB and p38 MAP Kinase Signaling Pathways,” Microbes and Infection 8, no. 4 (2006): 994–1005. 16513392 10.1016/j.micinf.2005.10.019

[102] I. M. Linhares , P. R. Summers , B. Larsen , P. C. Giraldo , and S. S. Witkin , “Contemporary Perspectives on Vaginal pH and Lactobacilli,” American Journal of Obstetrics and Gynecology 204, no. 2 (2011): 120.e1–120.e5. 10.1016/j.ajog.2010.07.010 20832044

[103] F. De Seta , R. Banco , A. Turrisi , et al., “Pelvic Inflammatory Disease (PID) From Chlamydia Trachomatis Versus PID From Neisseria Gonorrhea: From Clinical Suspicion to Therapy,” Giornale Italiano di Dermatologia e Venereologia: Organo Ufficiale, Societa Italiana di Dermatologia e Sifilografia 147, no. 5 (2012): 423–430. 23007248

[104] K. A. Green , S. M. Zarek , and W. H. Catherino , “Gynecologic Health and Disease in Relation to the Microbiome of the Female Reproductive Tract,” Fertility and Sterility 104, no. 6 (2015): 1351–1357. 26597627 10.1016/j.fertnstert.2015.10.010

[105] M. Champer , A. Wong , J. Champer , et al., “The Role of the Vaginal Microbiome in Gynaecological Cancer,” BJOG: An International Journal of Obstetrics & Gynaecology 125, no. 3 (2018): 309–315. 28278350 10.1111/1471-0528.14631

[106] E. K. Libby , K. E. Pascal , E. Mordechai , M. E. Adelson , and J. P. Trama , “ *Atopobium vaginae* Triggers an Innate Immune Response in an In Vitro Model of Bacterial Vaginosis,” Microbes and Infection 10, no. 4 (2008): 439–446. 18403235 10.1016/j.micinf.2008.01.004

[107] M. R. Zariffard , R. M. Novak , N. Lurain , B. E. Sha , P. Graham , and G. T. Spear , “Induction of Tumor Necrosis Factor–α Secretion and Toll‐Like Receptor 2 and 4 mRNA Expression by Genital Mucosal Fluids From Women With Bacterial Vaginosis,” Journal of Infectious Diseases 191, no. 11 (2005): 1913–1921. 15871126 10.1086/429922

