# Supplementary material for: Bacterial Infections Role in Gynecological Cancers Development: Narrative Review
Source: Cancer Rep (Hoboken). 2026 Apr 12;9(4):e70499. doi: 10.1002/cnr2.70499 (PMC13070870; doi:10.1002/cnr2.70499)
Supplement: Supplementary file 1 — Figure S1: An overview of the literature search strategy. [file CNR2-9-e70499-s001.docx]

Flow chart of the literature selection process in the present articles

-Excluded studies

-Review and congress abstract

-Studies not reporting

Excluded Studies

Title and abstract screening

Studies detected by the initial screening of databases

Google scholar

PubMed

Included studies

Full text

Duplication

Scopus

Figure-1 of Supporting Information: An overview of the literature search strategy
